# Molecular Tools for Detection and Identification of *Paracoccidioides* Species: Current Status and Future Perspectives

**DOI:** 10.3390/jof6040293

**Published:** 2020-11-18

**Authors:** Breno Gonçalves Pinheiro, Rosane Christine Hahn, Zoilo Pires de Camargo, Anderson Messias Rodrigues

**Affiliations:** 1 Laboratory of Emerging Fungal Pathogens, Department of Microbiology, Immunology, and Parasitology, Discipline of Cellular Biology, Federal University of São Paulo (UNIFESP), São Paulo 04023062, Brazil; brenogonpi@gmail.com (B.G.P.); zpcamargo1@gmail.com (Z.P.d.C.); 2 Laboratory of Mycology/Research, Faculty of Medicine, Federal University of Mato Grosso, Cuiabá, Mato Grosso 78060900, Brazil; rchahn@terra.com.br; 3 Federal University of Mato Grosso, Júlio Muller University Hospital, Mato Grosso 78048902, Brazil; 4 Department of Medicine, Discipline of infectious Diseases, Federal University of São Paulo (UNIFESP), São Paulo 04023062, Brazil

**Keywords:** molecular diagnostics, *Paracoccidioides brasiliensis*, *Paracoccidioides lutzii*, paracoccidioidomycosis, epidemiology, diagnosis, endemic mycosis, systemic mycosis

## Abstract

Paracoccidioidomycosis (PCM) is a mycotic disease caused by the *Paracoccidioides* species, a group of thermally dimorphic fungi that grow in mycelial form at 25 °C and as budding yeasts when cultured at 37 °C or when parasitizing the host tissues. PCM occurs in a large area of Latin America, and the most critical regions of endemicity are in Brazil, Colombia, and Venezuela. The clinical diagnosis of PCM needs to be confirmed through laboratory tests. Although classical laboratory techniques provide valuable information due to the presence of pathognomonic forms of *Paracoccidioides* spp., nucleic acid-based diagnostics gradually are replacing or complementing culture-based, biochemical, and immunological assays in routine microbiology laboratory practice. Recently, taxonomic changes driven by whole-genomic sequencing of *Paracoccidioides* have highlighted the need to recognize species boundaries, which could better ascertain *Paracoccidioides* taxonomy. In this scenario, classical laboratory techniques do not have significant discriminatory power over cryptic agents. On the other hand, several PCR-based methods can detect polymorphisms in *Paracoccidioides* DNA and thus support species identification. This review is focused on the recent achievements in molecular diagnostics of paracoccidioidomycosis, including the main advantages and pitfalls related to each technique. We discuss these breakthroughs in light of taxonomic changes in the *Paracoccidioides* genus.

## 1. Paracoccidioidomycosis: An Overview

*Paracoccidioides brasiliensis* and related species are the agents of paracoccidioidomycosis (PCM), a systemic mycosis of humans and animals. The disease is acquired following the inhalation of *Paracoccidioides* spp. propagules (e.g., conidia) from contaminated soil in endemic areas of Latin America [1,2]. Therefore, pulmonary involvement is present in nearly 90% of individuals. PCM is classified as an infection or disease, and the primary clinical forms are described as follows: (i) the acute or subacute form (juvenile), which presents rapid progression and can be considered severe due to high lethality rates in children and adolescents; (ii) the chronic form (adult), uni or multifocal, which can be mild, moderate or severe and presents slower progression, being responsible for most cases of PCM (74 to 96%); and (iii) the residual form or sequelae, which are clinical manifestations of anatomical and functional changes observed after PCM treatment [1,3,4].

From an epidemiological perspective, PCM has a substantial public health impact and is frequently associated with poverty [5]. Nowadays, data from Latin American areas with stable endemicity such as Brazil and Colombia suggest that the incidence ranges between 1 and 4 cases per 100,000 inhabitants per year [4,6,7], while annual incidence rates as high as 9–40 cases/100,000 inhabitants can be reached in hyperendemic areas of Brazil (e.g., Rondônia and Mato Grosso states). Brazil alone accounts for more than 80% of the total cases reported in the literature [1,8].

The latest estimates are that mortality associated with PCM varies between 6.1% [9] and 7.6% [10], ranking it as the eighth-highest among chronic infections caused by eukaryotic pathogens, ahead of leaving leishmaniasis [7]. Even though the numbers are large, the disease is not subject to compulsory notification, and it is not yet registered as a neglected tropical disease by the World Health Organization (WHO), a status that could benefit PCM research [5].

The taxonomy of PCM agents has undergone several changes since the discovery of the disease at the beginning of the last century, driven first by the great morphological diversity [11,12,13] and deviating genetic features of some isolates. Such modifications have intensified in recent years by introducing molecular trait methods for the fungus’ genetic characterization, such as multilocus sequencing analysis [14,15,16,17] or whole-genome sequencing analysis [18,19].

**Figure 1:**
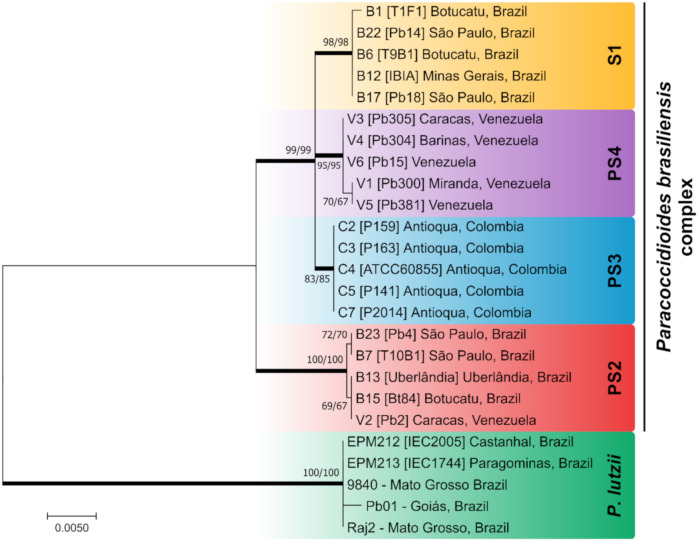
Phylogenetic tree inferred using the maximum likelihood method and Kimura 2-parameter model of the partial sequences of the immunodominant antigen GP43, ADP ribosylation factors (ARF), and tubulin alpha-1 chain (TUB1) of *Paracoccidioides* isolates. The *P. brasiliensis* complex is composed of the cryptic species S1, PS2, PS3, and PS4. *Paracoccidioides lutzii* appear as a divergent genetic group, apart from the *P. brasiliensis* complex. Numbers close to the branches represent bootstraps values (maximum likelihood/neighbor joining, respectively). Bootstraps higher than 80 based on 1000 replications are represented in bold branches. Sequences were described previously by Matute et al. [14], Teixeira et al. [15], and Hahn et al. [20].

Recent studies of molecular phylogeny have clarified species boundaries within clinical isolates of *Paracoccidioides* and provided strong genetic support for the existence of at least four phylogenetic molecular siblings, nested in a species complex named *P. brasiliensis* complex, in addition to a new species called *P. lutzii* (Figure 1) [17].

**Figure 2:**
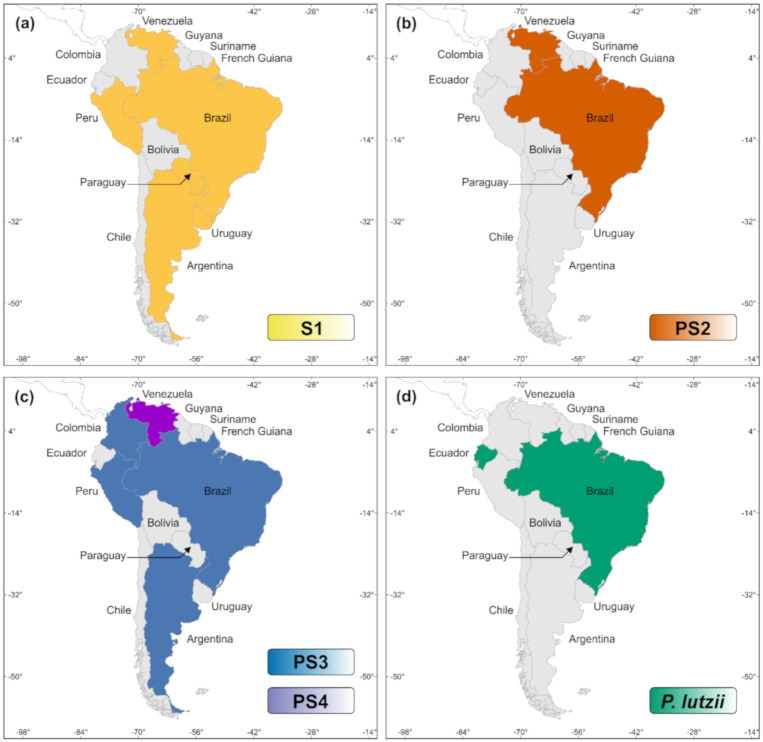
Distribution patterns of *Paracoccidioides* species in South America, based on epidemiological reports in the literature [19,23,24,27,28]. (**a**) *P. brasiliensis sensu stricto* (S1); (**b**) *P. americana* (PS2); (**c**) *P. restrepiensis* (PS3), and *P. venezuelensis* (PS4); (**d**) *P. lutzii*.

The *P. brasiliensis* complex includes the classical *P. brasiliensis* (S1, along with clusters S1a and S1b), phylogenetic species 2 (PS2), phylogenetic species 3 (PS3), and phylogenetic species 4 (PS4) [14,21,22]. Most of the molecular siblings occur in sympatry, as illustrated by S1 and PS2, showing a clear overlapping distribution in a vast area of South America. Phylogenetic species S1a and S1b are predominantly found in South America, especially in southeastern and southern Brazil, Argentina, and Paraguay. The PS2 group has sporadic distribution and is less frequently reported, with human cases reported thus far in Venezuela and southeast Brazil. Remaining genetic clusters such as PS3 and PS4 are less frequent PCM agents, and isolated cases have been found in Colombia and Venezuela, respectively. Recently, sparse cases related to PS3 have been found outside Colombia, in Argentina, Peru, and Brazil (Figure 2) [19,23,24]. *P. lutzii*, on the other hand, encompasses a single species and is predominantly distributed in the Midwest and Amazon regions of Brazil and Ecuador (Figure 2) [25,26]. The natural habitat of *P. lutzii* remains to be elucidated, as this species has not yet been isolated from armadillos [27,28].

The recent study of Turissini et al. [17] elevated the molecular siblings embedded in the *P. brasiliensis* complex to species level. Therefore, S1, PS2, PS3, and PS4 are named *P. brasiliensis sensu stricto*, *P. americana*, *P. restrepiensis*, and *P. venezuelensis*, respectively. However, the taxonomic argument’s strength should consider interrelated areas of classification, nomenclature, and identification to reflect phylogeny and evolution. Therefore, such modifications proposed for *Paracoccidioides* spp. are still a matter of debate since they are barely suitable for a polyphasic system. The polyphasic taxonomy [29] or the “consilient taxonomy” [30,31] approach used in nomenclature and systematic classification of a novel fungus should incorporate a combination of multiple, independent datasets such as phenotypic and genotypic data, as a means of delimiting species boundaries. Moreover, when delimitating medically relevant fungi, the need for modification in the taxonomy usually translates into differences related to clinical manifestations, differential virulence, and/or differential sensitivity to antifungal agents used for treatment [32]. Accordingly, in this review, we designate *P. brasiliensis* as representing a species complex and refer to it as the *P. brasiliensis* species complex.

There is no scientific evidence of whether different species cause different clinical manifestations. Previous data have shown a remarkable overlap of clinical symptoms of patients infected with various members of the *P. brasiliensis* complex or even between the *P. brasiliensis* complex and *P. lutzii* [33,34,35]. On the other hand, there is strong evidence from a seroepidemiological investigation that the diagnosis can be impaired when using specific antigenic preparations derived from *Paracoccidioides* spp., suggesting the need for regional antigen formulations to overcome this gap [36].

Therefore, species boundaries have a profound impact on the diagnosis of the supposed etiologic agent, affecting the ability to recognize different *Paracoccidioides* spp., which can influence the effectiveness of communication between the diagnostic laboratory technicians and the medical staff.

## 2. The Laboratory Diagnosis of PCM

**Figure 3:**
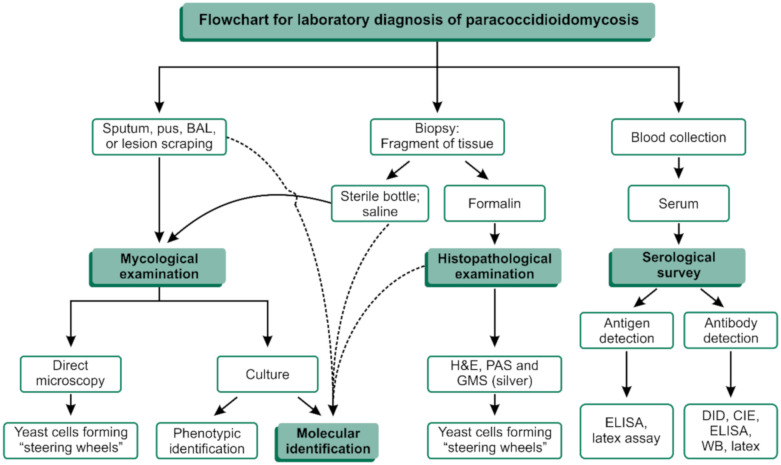
Flowchart for laboratory diagnosis of paracoccidioidomycosis (PCM). BAL: Bronchoalveolar lavage; H&E: Hematoxylin and eosin staining; PAS: Periodic acid–Schiff; GMS: Gomori methenamine silver; ELISA: enzyme-linked immunosorbent assay; DID: double immunodiffusion; CIE: counterimmunoelectrophoresis reaction; WB: Western blot.

**Figure 4:**
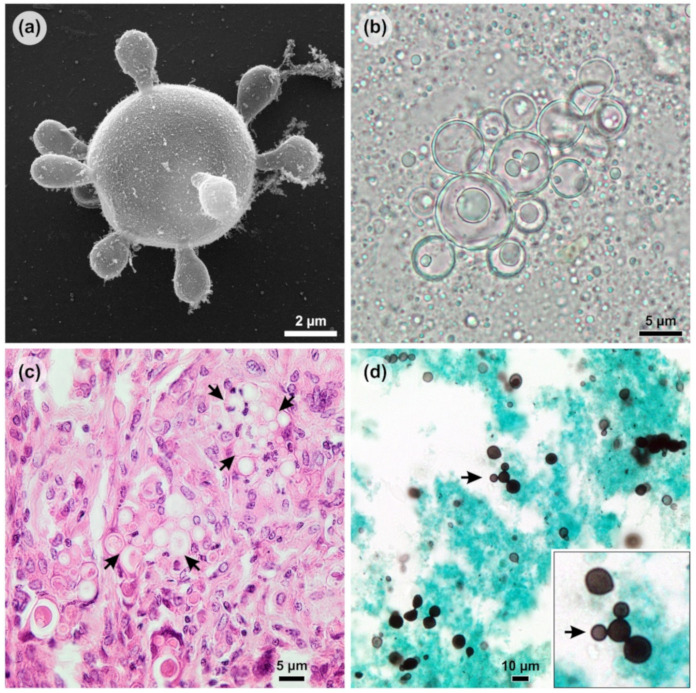
Morphological aspects of *Paracoccidioides* yeast. (**a**) *Paracoccidioides lutzii* showing multiple buds with a “steering wheel” shape in vitro (scanning electron microscopy); (**b**) The positive direct mycological examination of pus showing large yeasts (5–15 µm) that have a thick, birefringent cell wall with single or multiple buds; (**c**) *Paracoccidioides* spp. in tissue (arrows) stained by hematoxylin and eosin (HE); (**d**) *Paracoccidioides* spp. in tissue stained by Grocott’s methenamine silver (GMS) showing a “Mickey Mouse” shape (arrow).

The classical PCM diagnosis combines clinical evaluation and additional laboratory investigations, including routine culture-based, biochemical, and immunological assays in microbiology laboratories (Figure 3). The reference method for the diagnosis of PCM is established by the finding of fungal elements suggestive of *Paracoccidioides* spp. in microbiological examinations of sputum or other clinical specimens such as skin scrapings or lymph node aspiration material after 10% KOH clarification with or without calcofluor [37]. The positive direct mycological examination of large yeasts (5–15 µm) that have a thick, birefringent cell wall with single or multiple buds (Figure 4b). These multiple buds have a “steering wheel” or “Mickey Mouse” shape and are considered pathognomonic findings in the diagnosis of PCM [37] (Figure 4).

Culture-based and biochemical methods are available for the isolation and characterization of *Paracoccidioides* [37]. In vitro cultivation from clinical material such as sputum or tissue fragments is highly recommended and presents an isolation success between 86% and 100%. However, *Paracoccidioides* is a fastidious microorganism and will take an average of two to three weeks to start its development on Sabouraud dextrose agar from room temperature to 37 °C (Figure 3) [37].

Anatomopathological examination is a useful tool for detecting the pathogen in tissues, as it allows us to identify the etiological agent down to the genus level in 95% of cases [38]. The fungus’s pathognomonic structures can be visualized in its yeast-like phase by experienced observers and accurate observation after routine staining such as with hematoxylin and eosin (HE) (Figure 4c). Nevertheless, the use of specific stains, such as PAS (periodic acid–Schiff) and silver-based stains, such as Grocott’s methenamine silver (GMS) (Figure 4d), must be included to improve detection.

Specific serological tests are of great importance in the presumptive diagnosis and prognosis of the disease, allowing the physician to evaluate and monitor the patient’s response to the treatment. In this scenario, the routine use of double immunodiffusion (DID) stands out, which allows for the quantitative detection of circulating antibodies in PCM [39,40]. Immunodiagnostics also include techniques such as counterimmunoelectrophoresis reaction (CIE) [41], enzyme-linked immunosorbent assay (ELISA) [42], latex agglutination assay [43], and immunoblotting [44], which are available from different reference services in Brazil (Figure 3). These tests usually employ a 43 kDa glycoprotein (GP43) as the *P. brasiliensis* complex’s primary antigen to detect circulating antibodies and have sensitivity between 85% and 100% [38]. Concerning serology tests, *P. lutzii*, for instance, has a striking antigenic variation, and since most serologic tests were developed with *P. brasiliensis* complex antigens, *P. lutzii*-infected hosts may have false-negative results [40]. Nevertheless, false-positive reactions can occur in patients with histoplasmosis, lobomycosis, and more rarely, aspergillosis. For a complete review of the serology of PCM, refer to Camargo (2008) [38].

As an alternative to classical laboratory diagnosis, molecular assays can be used for *Paracoccidioides* spp. aiming at different strategies, such as detection and identification. In this scenario, molecular-based assays provide the fastest and most accurate results about the infection, reaching species level more efficiently [21]. The present review provides basic information about PCM diagnostics including the main advantages and drawbacks related to these techniques. The significant developments in the molecular diagnosis and characterization of *Paracoccidioides* spp. are discussed in light of the recent changes in the taxonomy, to determine the direction that new molecular tools should follow. The literature search criteria utilized for the preparation of this article are identified in the Supplementary Material (Table S1).

## 3. Molecular Diagnosis: Where Do We Stand?

Molecular assays are based on the detection of biomarkers such as DNA, RNA, and gene products from etiologic agents using techniques and/or equipment specifically developed to do so [45]. During the development of molecular techniques, scientists face a wide range of challenges, such as the inherent biology of the fungi (e.g., the presence of a rigid cell wall) and sample preparation, to cite a few. Though challenges exist, they are often overcome by several molecular techniques available to detect diverse fungi, chosen according to different necessities [46]. Considering *Paracoccidioides* spp., several molecular assays have been used for the detection of this pathogen from soil samples in rural and urban environments and for detection in aerosols, making ecological studies and geographical tracking easier, as well shedding light on fungal microenvironments [47,48,49,50]. Unfortunately, the same may not be valid for clinical routine, where tests have strict requirements for use in point-of-care testing or bedside testing.

Many variables should be considered when designing molecular assays: the purpose, equipment and reagent costs, maintenance requirements, need for operational expertise, clinical specimen type, execution speed, specificity and sensitivity (approaching 100%), positive and negative predictive values, output, post-assay data manipulation, or any other post-execution needed [51,52,53,54,55,56]. Detection directly from clinical specimens is desired, but in the medical mycology field, early-stage detection is infrequent, mainly when compared to virology and bacteriology fields [57,58,59]. In the case of *Paracoccidioides* alone, very few standardized technologies are available for use in clinical routine or to detect/identify the fungus in clinical specimens other than the culture-based and biochemical methods. Extra care has to be taken regarding *Paracoccidioides* and its recently described cryptic species, which are not always considered in experimental design [14,22].

PCR-based techniques are the most common in medical mycology due to their versatility and ability to target and amplify nucleic acids from isolated strains or directly from clinical samples such as sputum, biopsy and bronchoalveolar lavage (BAL) material, cerebrospinal fluid (CSF), blood, etc., using fast, inexpensive, and widely available equipment [46,52,60,61,62]. On the other hand, there are difficult hurdles to overcome regarding PCR usage, for instance, suspicion about the etiologic agent due to the absence of screening or panfungal tests and small amounts of fungal genetic material from clinical samples [63].

**Figure 5:**
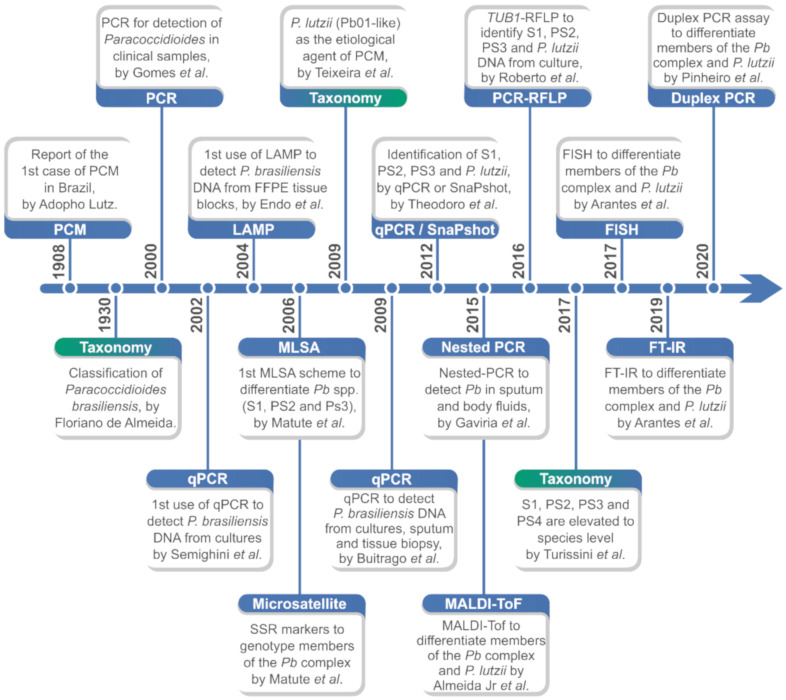
Major developments in the identification and molecular/proteomic characterization of *Paracoccidioides* species. PCM: paracoccidioidomycosis; *Pb*: *Paracoccidioides brasiliensis*; qPCR: quantitative real-time polymerase chain reaction; LAMP: loop-mediated isothermal amplification; FFPE: formalin-fixed paraffin-embedding; MLSA: multilocus sequence analysis; SSR: single sequence repeats; Pb: *Paracoccidioides brasiliensis*; SnaPshot: single-nucleotide polymorphism (SNP) genotyping; MALDI-ToF: matrix-assisted laser desorption/ionization time-of-flight mass spectrometry; PCR-RFLP: polymerase chain reaction-restriction fragment length polymorphism; *TUB1*: tubulin alpha-1 chain; FISH: fluorescence in situ hybridization; FT-IR: Fourier-transform infrared spectroscopy.

Judging from PCM diagnostics, an impressive number of PCR-based techniques can be found for species identification and/or differentiation (Figure 5). However, most of them are not widely used in clinical routines due to their prices, design characteristics, and implementation requirements, not to mention their limitations. The majority of these tests were also developed before discovering *P. lutzii* and may not consider this agent in their scope, while other tests were designed before the full description of the agents included in the *P. brasiliensis* complex [64,65]. Some techniques have continuously been revised and adapted to meet the changing needs of researchers and clinicians, mainly after the changes this genus experienced with whole-genome sequencing studies and the questions raised about species boundaries in the last decade [17,18,22,65]. Below, molecular techniques with diagnostic purposes are reviewed (Figure 5).

### 3.1. Internal Transcribed Spacer as a Barcoding Marker in Paracoccidioides

Beginning in 2003, the DNA barcoding initiative firmly demonstrated that most living species could be distinguished by a short stretch of DNA sequences [66]. In fungi, the sequencing of ribosomal 18S or 28S regions can be a useful tool for genus identification. Otherwise, there is enough variability in the internal transcribed spacer (ITS) region and D1/D2 domains to allow identification down to the species level and below. In the attempt to standardize molecular identification, a multinational research consortium chose ITS as the universal DNA barcode marker for fungal identification. This decision was supported by the available taxonomic resolution provided, as well as the regional characterization in sequence databases such as GenBank [67,68].

According to the pathogens assessed by the International Society for Human and Animal Mycology-ISHAM, by Irinyi et al. [69], DNA barcoding through ITS sequencing alone (i.e., ITS1/2 + 5.8 s) is perfectly possible for generic identification of *Paracoccidioides* isolates since they account for a moderate genetic diversity range of 1.01–1.5% in 17 polymorphic sites, of the 8 isolates analyzed. The ISHAM-ITS database proposes a workflow to identify unknown fungal isolates and should be sufficient in queries where identity surpasses 98.5% [69]. Primers used to amplify and sequence the ITS region in *Paracoccidioides* are those designed by White et al. and named ITS1 and ITS4 [70].

**Figure 6:**
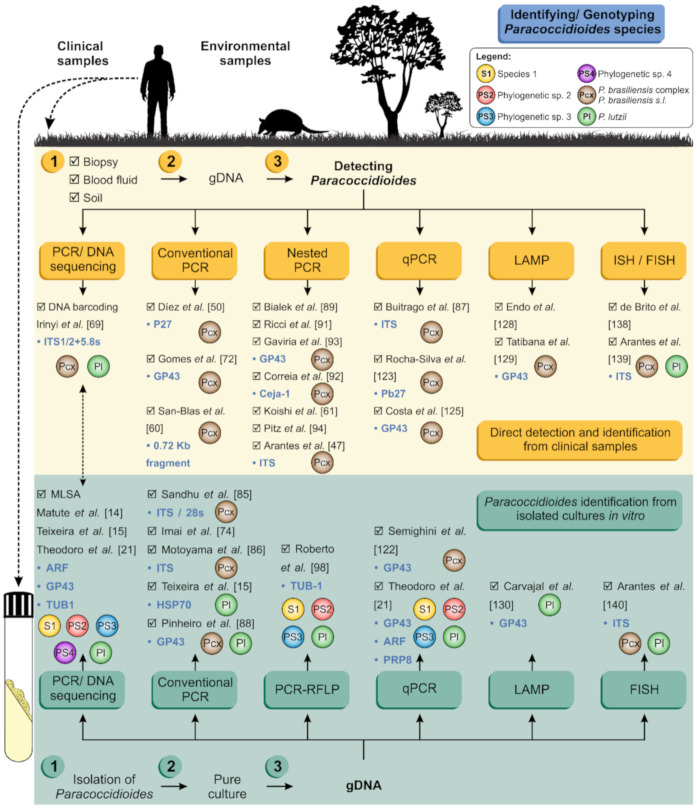
Schematic representation of *Paracoccidioides* spp. molecular detection/identification pipeline, directly from clinical and/or environmental samples (yellow panel) or from gDNA extracted from cultured isolates (green panel). Breakthroughs are listed, as well as their targeted genes and species discrimination power. PCR, polymerase chain reaction; RFLP, restriction fragment length polymorphism; qPCR, quantitative real-time polymerase chain reaction; FISH, fluorescent in situ hybridization; LAMP, loop-mediated isothermal amplification.

In the past, phylogenetic analyses of the 18S rRNA to distinguish between *Paracoccidioides* spp. and *Blastomyces dermatitidis* were proposed, since disease differentiation could be problematic. Both species were grouped inside the Onygenales order, and although they produce similar symptoms, therapy can differ [71]. As the ITS region and GP43 gene became available, the number of PCR-based methods for *Paracoccidioides* identification increased [72,73,74]. The ITS region offers enough polymorphisms for *Paracoccidioides* genus identification, as it does for other fungal species [74,75,76]. Any doubts were overcome later by Hebeler-Barbosa et al. [77], through evaluation of the ITS region and PbGP43 gene in isolates with different origins, such as armadillos and humans. Analyses confirmed similarity between both sample types, though isolates were clustered in two separate groups, not necessarily coincident between the chosen genes or between ITS and RAPD markers. Those results suggested that PbGP43 would be useful alongside ITS to carry out further studies of the *P. brasiliensis* complex [77]. To date, ITS barcoding allows differentiation between the *P. brasiliensis* complex and *P. lutzii* but has no discriminatory power regarding all four species contained in the *P. brasiliensis* complex (S1, PS2, PS3, and PS4), so the use of secondary barcoding markers (e.g., GP43) needs to be further discussed among members of the scientific community (Figure 6).

### 3.2. Multilocus Sequence Analysis (MLSA)

Multilocus sequence analysis (MLSA) is currently a widely used method to achieve a higher resolution of the phylogenetic relationships of any species within one or more genera. MLSA is based on multilocus sequence typing (MLST), which was first introduced by Maiden et al. in 1998 as a microbial typing method for epidemiological and population genetic studies of pathogenic bacterial species [78]. Usually, 3–5 genes with good quality are chosen to provide a scheme considering the possibility of primer design, quality of sequences available, length of fragments considered, and high-quality sequence alignment [79]. In *Paracoccidioides* spp., choosing schemes might be even more challenging since mitochondrial loci are not aligned across all species but seem to correspond to geographical location [16].

The first MLSA scheme for *Paracoccidioides* was proposed by Matute et al. [14] comprised of five nuclear coding genes, including chitin synthase (CHS2), β-glucan synthase (FKS), α-tubulin (TUB), ADP-ribosylation factor (ARF), and PbGP43 (GP43). This scheme separated *P. brasiliensis* complex members into three distinct groups, namely S1, PS2, and PS3 [80]. As this test failed to yield fragments for Pb01-like isolates, Carrero et al. [81] proposed a new scheme involving hydrophobin 5′ and 3′ UTRs (HYD1), heat-shock protein (HSP70), KEX, and an internal transcribed spacer (ITS), hypothesizing these isolates would belong to a different species, later named *P. lutzii*. Teixeira et al. [15] analyzed 13 loci, including the ones cited above. They managed to differentiate S1, PS2, PS3, and *P. lutzii*, with the combination of GP43, ARF, β-TUB, and HSP70 providing better results for species delineation. However, the scheme was not completed until the report of *P. venezuelensis* (PS4) by Teixeira et al. [22]. Therefore, MLSA is the only method that allows the recognition of five *Paracoccidioides* species. However, amplification followed by sequencing is possible only when using DNA extracted from pure cultures, which limits the application of MLSA for detection (Figure 1).

### 3.3. Conventional Polymerase Chain Reaction

The polymerase chain reaction (PCR) technique was developed in 1985 by Kary B. Mullis and allowed scientists to easily make unlimited copies of DNA from just one original strand [82]. This monumental scientific discovery was quickly incorporated into the molecular diagnosis scenario aiming for the detection of nucleic acids of the supposed pathogen as markers of infection.

PCR is a widespread technology that relies on equipment able to switch temperatures in pre-defined cycle sets, according to primers designed to accomplish specific tasks. It employs the heat-stable enzyme DNA polymerase to replicate the targeted genetic material within the whole DNA. Almost 15 years went by before PCR was first applied to PCM diagnosis [52,82,83].

Goldani et al. [84] reported the first cloning and sequencing of a species-specific DNA fragments from *P. brasiliensis sensu lato* (*s.l.*) related to the β-actin gene. Later, the rDNA region was revisited in search of fragments informative for *P. brasiliensis s.l.* Sandhu et al. [85] targeted a 200 bp fragment near the 5′ end of the 28S ribosomal gene. The protocol was completed by the characterization of amplicons using a species-specific hybridization probe. Using another gene, P27, Díez et al. [50] managed to detect *Paracoccidioides* DNA from artificially contaminated soil, aiming to launch a technique for ecological studies. Still searching for suitable candidates, Imai et al. [74] targeted the 5.8S region with a 418 bp DNA fragment tested against 29 isolates, while Motoyama et al. [86] did the same for both the 5.8S and 28S regions, with expected fragments of 649 bp and 496 bp, respectively, and tested them against a single *Paracoccidioides* isolate (Pb01).

All of the mentioned tests returned 100% specific results, leading to generic identification, but lacked sensitivity. A critical pitfall includes that the standardization nearly always occurred employing very few samples and using genetic material extracted from yeasts cultured in vitro, instead of detecting *Paracoccidioides* DNA directly from clinical samples such as sputum and biopsy. To overcome these problems, two genus-specific PCRs were developed and standardized to be run with clinical samples such as sputum and cerebrospinal fluid (CSF). Gomes et al. [72] used as a target the gene coding for the 43,000-Da antigen (GP43) to launch the first-ever PCR assay for use to analyze sputum for *Paracoccidioides* detection. San-Blas et al. [60] went further and tested sputum, cerebrospinal fluid, serum, and blood samples. Excellent agreement among clinical, serological, and molecular diagnoses was found for the sputum and cerebrospinal fluid samples, achieving a detection limit of 1 pg of *Paracoccidioides* DNA (Figure 6) [60,72].

Upon the description of *P. lutzii*, researchers began to understand a presumable diagnostic gap among the main *Paracoccidioides* species, creating the need for PCR assays able to detect *P. lutzii*. In this attempt, Teixeira et al. [15] developed a pair of *P. lutzii*-specific primers oriented to the HSP70 locus and achieved remarkable results using DNA from yeast cultured in vitro. Since the HSP70 assay was not conceived as a multiplex assay, it only solved part of the problem. Dias et al. [62] used a set of primers previously described for a real-time quantitative PCR test [87] targeting a 144 bp ITS fragment, trying to detect fungi from serum samples. Albeit limited, the results suggested that serum samples were not suited for PCR diagnosis of either species. Recently, Pinheiro et al. [88] explored the potential of a one-tube duplex PCR assay for the detection and identification of *Paracoccidioides* species. The duplex PCR assay was a fast, reliable, and easily implementable into a laboratory routine for recognizing members of the *P. brasiliensis* complex and *P. lutzii*, improving clinical diagnosis and epidemiological measures of PCM burden. Very few options are currently available concerning conventional PCR and the distinction between species using clinical samples (Figure 6).

### 3.4. Nested PCR and Semi-Nested PCR

Nested PCR is a modification of PCR designed to improve the sensitivity and specificity of the reaction as well as to reduce nonspecific amplification of the template DNA. Nested PCR involves the use of two primer sets and two successive PCRs. The first set of primers is conceived to anneal to sequences upstream from the second set of primers, and is used in an initial PCR reaction. After the first reaction, amplicons are used as a template for the second set of primers, and a second amplification step is performed, which generates a (shorter) sequence. Most of the nested PCR assays were developed to detect *Paracoccidioides* DNA during infection, and the main target of these assays is GP43 or the ITS region of the rDNA.

Bialek et al. [89] used a nested PCR assay to detect *P. brasiliensis s.l.* DNA in culture and tissue samples of *P. brasiliensis*-infected BALB/c mice. This nested PCR assay targeting the GP43 locus showed high sensitivity (0.5 fg) and specificity (100%). Likewise, Sano et al. [90] applied two nested PCR assays to detect the GP43 and ITS region derived from paraffin-embedded tissue specimens. The nested PCR assays were sensitive and specific, detecting *Paracoccidioides* DNA even in tissue samples with a small number of yeast cells. Later, Ricci et al. [91] proposed a semi-nested PCR strategy followed by amplicon sequencing to support the genotyping of the infective *P. brasiliensis s.l.* strain directly from paraffin-embedded tissue samples. A strong correlation of histopathological findings with genotypes was reported, supporting its use for understanding the *P. brasiliensis s.l.* epidemiology. Correia et al. [92] proposed a two-step PCR specific for virulent strains of *P. brasiliensis s.l.*, using the Ceja-1 sequence as a potential biomarker. More recently, Gaviria et al. [93] reported a nested PCR assay targeting the GP43 locus with specificity and sensitivity rates of 100%, allowing the detection down to 1 fg of *P. brasiliensis* DNA (Figure 6).

Koishi et al. [61] developed a semi-nested PCR for PCM diagnosis. The first round of PCR was based on the universal primers ITS1 and ITS4 [70], whereas the second round included the new inner primer MJ03 in addition to ITS1. The semi-nested PCR was useful to investigate biopsies of five patients with oral lesions that resembled PCM, detecting as little as 0.25 pg of *P. brasiliensis s.l.* DNA [61]. Later, this semi-nested-PCR was modified and optimized by Pitz et al. [94] to be performed through a one-tube assay, reducing contamination but maintaining high sensitivity even when used sputum directly from patients (Figure 6).

### 3.5. Restriction Fragment Length Polymorphism and PCR-RFLP

Restriction fragment length polymorphism (RFLP) is a technique based on the restriction of whole DNA [95], using endonucleases targeting known polymorphisms and yielding fragments whose band pattern is observed after gel electrophoresis [96]. RFLP can be combined with a conventional PCR technique, so the enzyme restriction is set to occur after DNA amplification directly on the amplicon, increasing the number of copies on which endonucleases can act. Both techniques are inexpensive, provide robust results, and are adaptable for different throughputs [97].

RFLP was first applied to correlate genetic diversity with the band pattern generated by strains from different countries and backgrounds, enabling inferences about genetic relationships. Although the technique allowed for the observation of five different genotypes, leading years later to the proposal of the cryptic species in the *P. brasiliensis* complex and *P. lutzii*, the main obstacle found was precisely the differentiation among clinical isolates [97].

Roberto et al. [98] used PCR-RFLP for *Paracoccidioides* identification and differentiation, considering cryptic species (S1, PS2, and PS3) and *P. lutzii*. The α-tubulin (*TUB1*) gene was chosen because it had enough polymorphisms to support differentiation among the species included in the *P. brasiliensis* complex. It is an interesting assay since it reaches the cryptic species level, rare in clinical routine, with only a few reaction rounds. However, it requires well-characterized strains for species identification, and was proposed before discovering the PS4 group, so it usually clustered these PS4 isolates together with other genetic groups (Figure 6).

### 3.6. RAPD

The random amplified polymorphic DNA (RAPD) technique allows for the amplification of DNA fragments under low-stringency conditions. This method is possible because the reaction involves arbitrary primers [99]. Consequently, since prior knowledge of the target sequences is not needed to observe amplification products, this technique has been widely applied for multiple purposes in *Paracoccidioides* spp. RAPD is often compared to RFLP, since it is designed to recognize the variation among isolates that are invariant under RFLP, or to discriminate isolates according to their geographic origin [96]. Soares et al. [100] were the first to apply the technique to *Paracoccidioides* spp., obtaining two different clusters. Calcagno et al. [101] were the first to assign five different phylogenetic groups to their place of origin with better stringency, as corroborated later by RFLP. However, this assay failed to correlate clusters with pathological features [101]. One year later, Totti et al. [102] used five isolates to study genomic variation and reported the test was able to reveal very little genetic diversity among the isolates assessed.

RAPD was used as a typing method by Hahn et al. [103] to explore the diversity among clinical strains of *Paracoccidioides*. Since the isolates clustered into two major groups, the results contrasted with Calcagno’s five groups [101], mostly due to the astounding genetic diversity of Brazilian isolates. The grouping patterns did not enable correlation with drug susceptibility, patient age, sex, or occupation. However, the most puzzling isolates were from Mato Grosso state, all of which clustered in two groups. The ones belonging to group II better responded to treatment and were related to the Pb01-like group, which was later identified as *P. lutzii* [15].

Molinari-Madlum et al. [104] found some degree of correlation for virulence in experimentally infected mice using well-characterized strains, but this could not be reproduced by Motta et al. [105] concerning virulence for mice or human clinical samples. Sano et al. [106] compared isolates according to host, isolated from armadillos and humans, finding no genetic heterogeneity implied from human samples. As for atypical isolates, Hahn et al. [103,107] retrieved two from natural cases of PCM, meaning the etiologic agents did not present yeast-mycelial dimorphism. Both atypical strains could not be set apart from the typical strains, while one of the typical strains was separated from all the others, establishing a new branch. Batista Jr. et al. [108] provided initial evidence of different *Paracoccidioides* genotypes co-infecting the same patient by analyzing lesions located at different host sites, corroborating the findings of Sano et al. [106] for armadillos.

Although the discrimination power of RFLPs in genetic diversity studies has been well documented in *Paracoccidioides* [97], the limitations related to the routine use of RFLP have prompted studies with other types of molecular markers, such as those involved in RAPD, which provide faster results and are simpler to use. The RAPD technology is well suited for DNA fingerprinting in *Paracoccidioides*, although pitfalls include a certain lack of reproducibility due to mismatched annealing. Despite these caveats, the RAPD method has contributed to the molecular epidemiology of *Paracoccidioides* spp. in endemic areas and to other associations, it was designed before the introduction of distinct species, so its use in clinical routine can be compromised.

### 3.7. Microsatellite Markers

Microsatellite markers (short sequence repeats-SSRs) are tandem repeated oligonucleotides composed of more than one repeat, whose length can change according to the number of repeated units. They provide fairly accurate allele characterization when observed by sizing or sequencing of PCR products with good scalability for high-throughput analysis [109]. They were first described by Jeffreys et al. [110] and have been used for typing important fungal pathogens, such as *Candida* spp. and *Aspergillus* spp. [111,112]. The first report of a *Paracoccidioides* spp. SSR marker library came from Nascimento et al. [113] when searching for a correlation between genetic background and clinical manifestations. Although SSR patterns did not reveal anything about fungal virulence and clinical features, it was important to support further studies. Matute et al. [114] used a combination of five SSR markers to recognize phylogenetic species inside the *P. brasiliensis* complex, as soon as they were reported. The test successfully differentiated between S1 and PS2 but lacked discriminatory power when including PS3 isolates [114].

### 3.8. Transposable Element Amplification

Transposable elements (Trems) were first discovered and described by McClintock et al. [115]. Based on the vast genetic diversity in the *Paracoccidioides* genus, and the lack of assays able to differentiate species, Alves et al. [116] developed a PCR-based method for species-specific amplification of Trems, as targets for identification. Trems are estimated to comprise approximately 16% of the *P. lutzii* genome and 9% of *P. brasiliensis*, with the possibility to distinguish between cryptic species [117]. Previously described Mariner-like transposons were thus utilized for this purpose with DNA extracted from cultured strains. The test was able to distinguish between *P. lutzii* and *P. brasiliensis* by amplification of TremA-H, reinforcing its specificity with conventional HSP70 PCR amplification and microsatellite markers. The use of Trems is a reliable, cheap, and easily performed technique, but neither Trems nor microsatellites markers have been able to reveal differences in the *P. brasiliensis* complex alone [116,118].

### 3.9. Quantitative Real-Time PCR

The fluorescence-based quantitative real-time PCR (qPCR) is a more advanced method in which an intercalating dye or a hydrolysis-based probe is added and hybridizes with both primers. Therefore, the amount of the PCR product can be determined, in real-time, in a wide range of samples from numerous sources. It is particularly useful for molecular diagnostics and for investigating gene expression. Often abbreviated as qPCR, this method is sometimes also referred to as real-time PCR, or depending on the application, quantitative reverse-transcriptase PCR (both abbreviated to RT-PCR, which can be confusing). Compared to conventional PCR, qPCR does not rely on any downstream analysis such as electrophoresis or densitometry, and it is incredibly versatile, enabling multiple PCR targets to be assessed simultaneously. qPCR assays were first proposed by Higuchi et al. [119] and are available for diverse fungi, such as *Aspergillus* spp. [120] and Mucorales species [121], among others.

The first report of a qPCR assay for detection and quantification of *P. brasiliensis s.l.* DNA was by Semighini et al., using the GP43 locus as a target to develop a fluorescent probe [122]. The assay could detect as few as 10 copies of the target DNA sequence, providing high specificity and sensitivity, useful for diagnosing PCM [122]. Buitrago et al. [87] used molecular probes targeting the ITS region of *P. brasiliensis*, with sensitivity as low as 1 fg DNA. Twelve cultured strains from two confirmed patients, as well as sputum, tissue and blood samples, were used. This assay is remarkable because of its accurate results for sputum and tissue biopsies, promising to diagnose PCM outside its endemic area [87].

After speciation in *Paracoccidioides*, Theodoro et al. [21] were the first to consider *Paracoccidioides* spp. diversity during identification and to carry out comparisons between multilocus sequence analysis (MLSA) and single nucleotide polymorphism analysis (SNaPshot), finding good agreement. qPCR reactions were carried out using fluorescent resonance energy transfer probes (TaqMan probes) and primers targeting the genes GP43, ARF, and PRP8 intein. The main goal was to differentiate between *P. lutzii* and the cryptic species included in the *P. brasiliensis* complex, such as S1, PS2, and PS3. Though this study contributed significantly to knowledge of *Paracoccidioides* spp. dispersal across South America, it did not cover the PS4 group, which was described two years later. Furthermore, the classification of some reference isolates was not accurate, as was the case of B-339, known to be PS3 [18,64,98], but identified as S1.

The *Paracoccidioides* specific gene coding for the Pb27 protein was chosen as the qPCR assay target to detect *Paracoccidioides* DNA [123,124]. The results of the qPCR standardization showed high efficiency (100%), high specificity (100%), and a low detection limit (10 to 100 fg). The assay was later used to detect *Paracoccidioides* DNA in tissue and peripheral blood of a patient that presented positive serology but negative culture [124]. Another interesting application of this technique was quantifying fungal load in experimentally infected mice [125]. Though promising, the method cannot differentiate between living and dead cells, which can be a problem [125].

### 3.10. Loop-Mediated Isothermal Amplification

Loop-mediated isothermal amplification (LAMP) is an inexpensive single-tube technique developed by Notomi et al. [126] for the rapid detection and amplification of DNA in pathogens. LAMP exhibits high specificity and selectivity because four primers are designed to recognize six distinct regions on the target base sequence. It can be completed quickly due to the high amplification efficiency under isothermal conditions (usually ranging from 60 to 65 °C) without the thermal cycler used in PCR. Consequently, LAMP employs a *Bst* DNA polymerase, a 67 kDa *Bacillus stearothermophilus* DNA polymerase protein with a potent strand-displacement activity, which synthesizes a new DNA strand dissociating the hydrogen bond of the double-stranded template DNA by itself. Therefore, DNA can be synthesized at isothermal conditions, and the secondary structure of DNA does not inhibit the synthesis [78,79]. One of the most promising features of this technique is the execution speed of only one to three hours, which is much shorter than the four hours of pipeline time required for conventional PCR, and up to 18 h for a nested-PCR. It thus is a reliable, cost-effective diagnostic tool without the need for sophisticated technical apparatus [126,127].

LAMP assays have been applied for the detection of *Paracoccidioides* DNA, mainly targeting the GP43 gene. Endo et al. [128] developed a LAMP assay to detect *Paracoccidioides* DNA from paraffin-embedded tissue samples from humans and armadillos. Based on targeting the gene GP43, they found that the LAMP could detect up to 100 fg of *Paracoccidioides* DNA [128]. LAMP was later tested by Tatibana et al. in sputa from 18 patients [129] using the same assay developed by Endo et al. [128]. According to the authors, the LAMP assay had the advantage of speed and simplicity, since it amplified in less than four hours 11 of the 18 sputum samples tested, much faster than the classic diagnostic methods such as histopathological testing or biological material culture, and did not require sophisticated technical apparatus [129]. However, the LAMP assays described above were designed not considering *P. lutzii* as a biological species. To fill in this gap, Carvajal [130], developed a new set of primers for use in the LAMP technique to differentiate *Paracoccidioides* species, achieving 100% analytical sensitivity (Figure 6).

### 3.11. Fluorescence In Situ Hybridization

In situ hybridization (ISH) is a technique that allows for precise localization and detection of nucleic acid sequences within a histologic section or structurally intact cells. ISH probes can diffuse across the cell wall and plasmatic membrane of fungi, targeting for example the rDNA in multiple clinical specimens. Fluorescence in situ hybridization (FISH) is a variant of ISH that uses fluorescent probes that bind to parts of the genome. Although FISH was initially used to classify chromosomes [131], this technique has since been implemented in a wide range of applications, including the diagnosis of fungal infections [132]. FISH demands little sample preparation, enabling fast execution, even allowing quantification. On the other hand, ISH-based assays do not have the same sensitivity as other tests [133,134]. A peptide nucleic acid (PNA)/FISH is now commercially available for the identification of some fungal pathogens (e.g., *Candida* spp., *Aspergillus* spp., *Fusarium* spp., *Scedosporium* spp.), as well as Yeast Traffic Light FISH and QuickFISH (AdvanDX, Woburn, MA, USA) [135,136].

In situ hybridization was created by Buongiorno-Nardell et al. [137] in the 1970s, but the first experiments with *Paracoccidioides* were only carried out in 1999 by Brito et al. [138]. The authors described an ISH assay in which a 14-mer probe targeting the 5′ terminus of the 28S ribosomal RNA subunit gene was used to detect *P. brasiliensis s.l.* in lesions biopsied from the oral cavity of seven PCM patients and in guinea pig testes inoculated with a culture of *P. brasiliensis s.l.* isolated from soil (Ibiá strain). However, the probe detected only 2–3% of the fungal cells present in the examined tissues, discouraging its use for routine diagnostic purposes [138].

A versatile and elegant use of this technique was demonstrated by Arantes et al. [139], who developed two target-specific probes that were successfully hybridized with members of the *P. brasiliensis* complex or *P. lutzii* (HRP-probe/TSA-FISH for *P. brasiliensis* and Texas Red-probe/FISH for *P. lutzii*) [139]. The advantage of the FISH method is its sensitivity and specificity rates, which are similar to those of the well-established nested PCR technique [47]. Moreover, FISH allowed the visualization of infective fungal structures of *Paracoccidioides* spp. directly in the environmental samples [139]. Furthermore, Arantes et al. [140] tried to extend the use of FISH for clinical purposes. The main goal was to launch a versatile technique to detect and differentiate *Paracoccidioides* spp. in their saprophytic and parasitic forms. This assay showed no cross-reactivity, even though the fluorescent signal for the *P. lutzii* probe was lower than it was for *P. brasiliensis* [140].

### 3.12. Matrix-Assisted Laser Desorption Ionization Time-of-Flight Mass Spectrometry

Matrix-assisted laser desorption/ionization mass spectrometry with time-of-flight detection (MALDI-ToF MS) is a powerful analytical mass spectrometry technique that is easy to use, rapid, accurate, and cost-effective. Although used since the 1980s in biochemistry [141], MALDI-ToF MS first emerged as a potential tool for microbial identification and diagnosis in the mid-1990s [142] and only reached clinical microbiology laboratories in 2010 [143]. Based on the advantages described above, MALDI-ToF MS-based typing is suitable to become a first-line epidemiological tool.

MALDI-ToF MS-based identification of microorganisms requires the generation of mass spectra from whole-cell material or extracted intracellular content. Fungal mass patterns are derived from structural proteins such as ribosomal proteins or other abundant fungal proteins, which can be used as biomarkers for species recognition (the mass range acquisition is mainly *m*/*z* 2000 to 20,000). Fingerprints built for unknown organisms are then matched to known references in a database (in-house or commercial database). Once the samples are ready, species identification takes only a few minutes, and depending on the MALDI-ToF MS score, the genus and species identification for an organism can be achieved with great accuracy. Accurate identification depends on two elements: adequate spectrum quality and close database reference matches.

Judging from the fungal identification, the whole process needs to be run with analytes generated from pure cultured fungi, using intact cells or cell extracts. Thus far, the need to isolate the microorganism can be a bottleneck and add days to the identification process [144]. The database had a fair amount of success identifying yeasts of medical relevance such as *Candida* spp. and *Cryptococcus* spp., and several filamentous fungi [145,146,147].

Among dimorphic fungi, *Paracoccidioides* identification was first proposed by Almeida Jr et al. [148], aiming to standardize an assay with spectra obtained from proteins expressed by members of the *P. brasiliensis* complex and *P. lutzii* (Figure 6). No misidentifications were found among the 22 strains tested. Regardless of the lack of definitive data correlating each species to clinical features, the test did not differentiate cryptic species inside the *P. brasiliensis* complex, information that could be useful for epidemiological studies [33,34].

### 3.13. Fourier-Transform Infrared Spectroscopy

Fourier-transform infrared spectroscopy (FT-IR) involves an infrared light scanning through a sample, which can be a whole cell or its extracts, to generate fingerprint spectra specific to each fungus, since molecules absorb the light in different wavelengths [149]. The principle of the technique was described by Cooley and Turkey in 1965 [150]. It has already been used in the mycology field for species identification (e.g., *Cryptococcus* spp. and *Exophiala* spp.) [151,152]. Comparato Filho et al. [153] used the vibrational spectroscopy signatures of proteins, polysaccharides, and nucleic acids to differentiate *P. brasiliensis* and *P. lutzii* successfully. They could visualize the *P. brasiliensis* complex’s genetic diversity but could not define parameters for their differentiation. The test does not require extensive sample preparation, such as MALDI-ToF, cutting valuable time off the diagnostic pipeline. On the other hand, it has some challenging technical requirements, possibly affecting reproducibility [153].

## 4. Molecular Diagnosis: Where Do We Go Next?

Judging from all molecular and proteomic assays discussed here, only a few tests are suitable for investigating clinical samples aiming to detect *Paracoccidioides* species’ DNA during infection, and none of them are designed to discriminate among the cryptic species described over the last decade. This pitfall may be due to a series of factors that include the difficulty of collecting enough samples for standardization and validation, or related to the low fungal burden of samples such as blood samples [87]. Furthermore, the nature of clinical samples can affect the final result. This is the case of paraffin-embedded tissues, which usually contain nucleic acids that are severely degraded and produce small fragments of DNA, generally no longer than 300 bp [154]. Likewise, biopsies are often surrounded by granulomatous tissue as part of PCM pathogenesis, which might degenerate or distort yeast cells and their content. Therefore, applying molecular techniques in these types of samples may be tricky, so researchers should hand-pick the ones with attested quality for retrospective studies [90,155].

The primary clinical specimen applied in routine diagnosis is sputum and tissue biopsies, including paraffin-embedded tissues. Protocols using conventional PCR showed good performance on sputum but not in serum samples, with the lower limit of detection being 10 cell/mL and 1.1 pg, respectively [62,72]. As for the nested-PCR, the best result was impressively 1 fg of sensitivity for the detection of the fungus on sputum [93], and 0.25 pg when using tissue biopsies [61]. Application of qPCR revealed the test might work better on sputum than in blood samples [87], while the tissue detection would have no problem if available *Paracoccidioides* species’ DNA is greater than 10 fg [123]. These results encourage the detection of *Paracoccidioides* in clinical routine and highlight that obtaining an excellent clinical specimen is the first major step in maintaining diagnostic accuracy.

The vast majority of molecular-based assays are applied to *Paracoccidioides* that are isolated in cultures. ITS region and GP43 gene are the primary markers used for molecular techniques, both of which are part of nuclear DNA. The GP43 gene has been discussed for use as a secondary barcode marker [90,156]. Perhaps a better answer lies in mitochondrial genes with larger copy numbers, increasing sensitivity in molecular assays, which could help circumvent the low fungal DNA content of samples [16,157]. Additionally, the average time between the discovery of a given molecular-based technique (e.g., PCR in 1985) and its application in the molecular diagnosis of PCM exceeds a decade (~15 years), revealing the slow pace of incorporating technology in the diagnosis of this important mycosis. Although these studies provided many contributions to PCM and etiologic agent studies, most of them have intrinsic limitations that make their clinical use impracticable. Remarkably, most techniques described here cannot accompany taxonomic updates of *Paracoccidioides*, mainly for not including *P. lutzii* or not considering the speciation events in the *P. brasiliensis* complex [18].

With the advent of whole-genome sequencing and its availability to the scientific community, new diagnostic tests based on NGS methods can be an important alternative, both from a diagnostic and an epidemiological perspective. Finally, it is essential for researchers to develop assays that can be used for point-of-care testing (POCT or bedside testing) [158]. These improvements will facilitate the communication between researchers and clinicians, greatly reduce the time to obtain results, and likely positively influence patient management and outcomes.

## Supplementary Materials

The following are available online at https://www.mdpi.com/2309-608X/6/4/293/s1, Table S1: Summary of molecular diagnosis methods in paracoccidioidomycosis.
