# Supplementary material for: Molecular Tools for Detection and Identification of Paracoccidioides Species: Current Status and Future Perspectives
Source: J Fungi (Basel). 2020 Nov 18;6(4):293. doi: 10.3390/jof6040293 (PMC7711936; doi:10.3390/jof6040293)
Supplement: Supplementary file 1 [file jof-06-00293-s001.pdf]

Supplementary material

Supplementary material: Molecular diagnosis of paracoccidioidomycosis: current status and future perspectives

Breno Gonçalves Pinheiro[1], Rosane Christine Hahn[2], Zoilo Pires de Camargo[1,3], Anderson Messias Rodrigues[1,*]

[1] Laboratory of Emerging Fungal Pathogens, Department of Microbiology, Immunology, and Parasitology, Discipline of Cellular Biology, Federal University of São Paulo (UNIFESP), São Paulo, Brazil.
[2] Federal University of Mato Grosso, Faculty of Medicine, Laboratory of Mycology/Research, Cuiabá, Mato Grosso, Brazil, Federal University of Mato Grosso, Júlio Muller University Hospital, Mato Grosso, Brazil.
[3] Department of Medicine, Discipline of infectious Diseases, Federal University of São Paulo (UNIFESP), São Paulo, Brazil.
* Correspondence: amrodrigues.amr@gmail.com; Tel.: +55-1155764551 Ext. 1540.

1. Search strategy

A search of PubMed was undertaken in September 2020. The search terms used were: (1) *Paracoccidioides* AND molecular diagnosis; (2) Paracoccidioidomycosis AND molecular diagnosis; (3) *Paracoccidioides* AND PCR; (4) *Paracoccidioides* AND MLSA; (5) *Paracoccidioides* AND barcoding; (6) *Paracoccidioides* AND internal transcribed spacer; (7) *Paracoccidioides* AND restriction fragment length polymorphism; (8) *Paracoccidioides* AND restriction amplified polymorphic DNA; (9) *Paracoccidioides* AND loop-mediated isothermal amplification; (10) *Paracoccidioides* AND qPCR; (11) *Paracoccidioides* AND *in situ* hybridization; (12) *Paracoccidioides* AND MALDI-ToF; (13) *Paracoccidioides* AND FTIR; (14) *Paracoccidioides* AND transposable elements; (15) *Paracoccidioides* AND microsatellites, and 73-72-116-0-1-16-6-2-2-29-3-3-3-3-6 results were returned, respectively. These results were manually filtered to recover articles that describe new methods aiming to be used in the molecular diagnosis of paracoccidioidomycosis.

Search #	Terms	Period covered	N. of articles
1	*Paracoccidioides* AND molecular diagnosis	1986-2020	73
2	Paracoccidioidomycosis AND molecular diagnosis	1986-2020	72
3	*Paracoccidioides* AND PCR	1995-2020	116
4	*Paracoccidioides* AND MLSA	-	0
5	*Paracoccidioides* AND barcoding	2019-2020	1
6	*Paracoccidioides* AND internal transcribed spacer	1998-2020	16
7	*Paracoccidioides* AND restriction fragment length polymorphism	2000-2020	6
8	*Paracoccidioides* AND restriction amplified polymorphic DNA	1999-2020	2
9	*Paracoccidioides* AND loop-mediated isothermal amplification	2004-2020	2
10	*Paracoccidioides* AND qPCR	2004-2020	29
11	*Paracoccidioides* AND in situ hybridization	1999-2020	3
12	*Paracoccidioides* AND MALDI-ToF	2010-2020	3
13	*Paracoccidioides* AND FTIR	2013-2020	3
14	*Paracoccidioides* AND transposable elements	2009-2020	3
15	*Paracoccidioides* AND microsatellites	2000-2020	6

The articles described in the Supplementary Table S1 were included in this review.

Table S1: Summary of molecular diagnosis methods in paracoccidioidomycosis.

Technique	Identification[1]	Species[2]	Typing[3]	Sample	Sensitivity	Specificity	Target	Ref.[4]
PCR	Yes	Pcx	No	Culture	N/A	N/A	β-actin	[1]
PCR	Yes	Pcx	No	Culture	N/A	100%	ITS 28S	[2]
PCR	Yes	Pcx	No	Culture, soil armadillo tissue	3 pg	N/A	P27	[3]
PCR	Yes	Pcx	No	Culture	N/A	100%	ITS 5.8S	[4]
PCR	Yes	Pcx	No	Culture	N/A	N/A	ITS 5.8S 28S	[5]
PCR	Yes	Pcx	No	Sputum	10 cells/ml	N/A	GP43	[6]
PCR	Yes	Pcx	No	Sputum, CSF	10 pg	N/A	0.72 kb fragment	[7]
PCR	Yes	Pl	No	Culture	N/A	N/A	HSP70	[8]
PCR	Yes	Pl	No	Serum	1.1 pg/μl	N/A	ITS	[9]
Nested-PCR	Yes	Pcx	No	Tissue	N/A	N/A	GP43	[10]
Nested-PCR	Yes	Pcx	Yes	FFPE	N/A	N/A	GP43	[11]
Nested-PCR	Yes	Pcx	No	Culture, lymph nodes	N/A	N/A	Ceja-1	[12]
Semi-nested-PCR	Yes	Pcx	No	Biopsy	0.25 pg	100%	ITS	[13]
Semi-nested-PCR	Yes	Pcx	No	Sputum	2.5 pg	100%	ITS	[14]
Nested-PCR	Yes	Pcx	No	Culture, sputum, CSF, body fluids	1 fg	100%	GP43	[15]
Nested-PCR	Yes	*Paracoccidioides*	No	Soil	N/A	N/A	ITS	[16]
RFLP	No	*Paracoccidioides* (5 groups)	Yes	Culture	N/A	N/A	Whole-genome	[17]
PCR-RFLP	Yes	S1, PS2, PS3, Pl	Yes	Culture	N/A	N/A	TUB-1	[18]
RAPD	No	*Paracoccidioides* (2 groups)	Yes	Culture	N/A	N/A	Whole-genome	[19]
RAPD	No	*Paracoccidioides* (5 groups)	Yes	Culture	N/A	N/A	Whole-genome	[20]
RAPD	No	*Paracoccidioides* (2 groups)	Yes	Culture	N/A	N/A	Whole-genome	[21]
RAPD	No	*Paracoccidioides* (3 groups)	Yes	Culture	N/A	N/A	Whole-genome	[22]
RAPD	No	*Paracoccidioides*	Yes	Culture	N/A	N/A	Whole-genome	[23]

Technique	Identification[1]	Species[2]	Typing[3]	Sample	Sensitivity	Specificity	Target	Ref.[4]
		(2 groups)						
RAPD	No	*Paracoccidioides* (2 groups)	Yes	Culture	N/A	N/A	Whole-genome	[24]
RAPD	No	*Paracoccidioides* (2 groups)	Yes	Culture	N/A	N/A	Whole genome	[25,26]
RAPD	No	*Paracoccidioides*	Yes	Culture	N/A	N/A	Whole-genome	[27]
LAMP	Yes	Pcx	No	FFPE	100 fg	N/A	GP43	[28]
LAMP	Yes	Pcx	No	Sputum	N/A	N/A	GP43	[29]
LAMP	Yes	Pl	No	Culture	N/A	100%	GP43	[30]
qPCR	Yes	Pcx	No	Culture	N/A	100%	GP43	[31]
qPCR	Yes	Pcx	No	Culture, Sputum, tissue, serum	N/A	100% culture, 50% serum	ITS	[32]
qPCR	Yes	S1, PS2, PS3, Pl	No	Culture	N/A	N/A	GP43, ARF, PRP8	[33]
qPCR	Yes	*Paracoccidioides*	No	Culture, tissue, serum	10 fg	100%	PB27	[34]
qPCR	Yes	Pcx	No	Mice	2 fg	N/A	GP43	[35]
ISH	Yes	Pcx	No	Oral biopsies	N/A	N/A	ITS	[36]
ISH	Yes	Pcx, Pl	No	Aerosol	N/A	N/A	ITS	[16]
FISH	Yes	Pcx, Pl	No	Slides	N/A	N/A	ITS	[37]
MALDI-ToF	Yes	Pcx, Pl	Yes	Culture	N/A	N/A	-	[38]
FT-IR	Yes	Pcx, Pl	Yes	Culture	N/A	N/A	-	[39]
Transposable Elements	Yes	Pcx, Pl	Yes	Culture	N/A	N/A	Mariner Transposon	[40]
Microsatellites	No	*Paracoccidioides*	Yes	Culture	N/A	N/A	Whole-genome	[41]
Microsatellites (SSR)	No	*Paracoccidioides* (3 groups)	Yes	Culture	N/A	N/A	Whole-genome	[42]
Microsatellites (SSR)	No	*Paracoccidioides* (4 groups)	Yes	Culture	N/A	N/A	Whole-genome	[43]
MLSA	Yes	S1, PS2, PS3	Yes	Culture	N/A	N/A	CHS2, FKS, TUB, ARF, PBGP43	[44]
MLSA	Yes	S1, PS2, PS3, Pl	Yes	Culture	N/A	N/A	HYD1, HSP, KEX, ITS	[45]

Technique	Identification[1]	Species[2]	Typing[3]	Sample	Sensitivity	Specificity	Target	Ref.[4]
MLSA	Yes	S1, PS2, PS3, Pl	Yes	Culture	N/A	N/A	GP43, ARF, β-TUB, HSP70	[8]
ITS Barcoding	Yes	Pcx	Yes	Culture	N/A	N/A	ITS	[46,47]
ITS Barcoding	Yes	Pcx, Pl	Yes	Culture	N/A	N/A	ITS, GP43	[48]

[1]Does the method allow identification? [2]Speciation level allowed; [3]Does the method allows for molecular typing? [4]Reference. PCM: paracoccidioidomycosis; Pcx: *P. brasiliensis* complex = *P. brasiliensis sensu lato*; Pl: *P. lutzii*; CSF: cerebrospinal fluid; PCR: polymerase chain reaction; qPCR: quantitative real-time polymerase chain reaction; LAMP: loop-mediated isothermal amplification; FFPE: formalin-fixed paraffin-embedding; MLSA: multilocus sequence analysis; SSR: single sequence repeats; Pb: *Paracoccidioides* brasiliensis; SnaPshot: single-nucleotide polymorphism (SNP) genotyping; MALDI-ToF: matrix-assisted laser desorption/ionization time-of-flight matrix-assisted laser desorption/ionization time-of-flight mass spectrometry; PCR-RFLP: polymerase chain reaction-restriction fragment length polymorphism; TUB1: tubulin alpha-1 chain; FISH: fluorescence *in situ* hybridization; FT-IR: Fourier-transform infrared spectroscopy; ITS: Internal transcribed spacer; GP43: 43-kDa glycoprotein; ARF: ADP-ribosylation factor ; PRP8: Pre-mRNA processing 8; P27: 27-kDa protein; HSP70: 70kDa-heat-shock protein; β-actin: Beta-actin; CHS2: Chitin synthase; FKS: β-glucan synthase; TUB: tubulin; HYD1: Hydrophobin 1; KEX: Kex protein gene; N/A: not available.

References

1. Goldani, L.Z.; Maia, A.L.; Sugar, A.M. Cloning and nucleotide sequence of a specific DNA fragment from *Paracoccidioides brasiliensis*. *J Clin Microbiol* **1995**, *33*, 1652-1654, doi:10.1128/jcm.33.6.1652-1654.1995.
2. Sandhu, G.S.; Aleff, R.A.; Kline, B.C.; da Silva Lacaz, C. Molecular detection and identification of *Paracoccidioides brasiliensis*. *J Clin Microbiol* **1997**, *35*, 1894-1896, doi:10.1128/jcm.35.7.1894-1896.1997.
3. Díez, S.; Garcia, E.A.; Pino, P.A.; Botero, S.; Corredor, G.G.; Peralta, L.A.; Castaño, J.H.; Restrepo, A.; Mcewen, J.G. PCR with *Paracoccidioides brasiliensis* specific primers: potential use in ecological studies. *Rev Inst Med Trop Sao Paulo* **1999**, *41*, 351-358.
4. Imai, T.; Sano, A.; Mikami, Y.; Watanabe, K.; Aoki, F.H.; Branchini, M.L.; Negroni, R.; Nishimura, K.; Miyaji, M. A new PCR primer for the identification of *Paracoccidioides brasiliensis* based on rRNA sequences coding the internal transcribed spacers (ITS) and 5 x 8S regions. *Med Mycol* **2000**, *38*, 323-326, doi:10.1080/mmy.38.4.323.326.
5. Motoyama, A.B.; Venancio, E.J.; Brandao, G.O.; Petrofeza-Silva, S.; Pereira, I.S.; Soares, C.M.; Felipe, M.S. Molecular identification of *Paracoccidioides brasiliensis* by PCR amplification of ribosomal DNA. *J Clin Microbiol* **2000**, *38*, 3106-3109.
6. Gomes, G.M.; Cisalpino, P.S.; Taborda, C.P.; de Camargo, Z.P. PCR for diagnosis of paracoccidioidomycosis. *J Clin Microbiol* **2000**, *38*, 3478-3480.
7. San-Blas, G.; Niño-Vega, G.; Barreto, L.; Hebeler-Barbosa, F.; Bagagli, E.; Olivero de Briceño, R.; Mendes, R.P. Primers for clinical detection of *Paracoccidioides brasiliensis*. *J Clin Microbiol* **2005**, *43*, 4255-4257, doi:10.1128/jcm.43.8.4255-4257.2005.
8. Teixeira, M.M.; Theodoro, R.C.; de Carvalho, M.J.A.; Fernandes, L.; Paes, H.C.; Hahn, R.C.; Mendoza, L.; Bagagli, E.; San-Blas, G.; Felipe, M.S.S. Phylogenetic analysis reveals a high level of speciation in the *Paracoccidioides* genus. *Mol Phylogenet Evol* **2009**, *52*, 273-283, doi:http://dx.doi.org/10.1016/j.ympev.2009.04.005.
9. Dias, L.; de Carvalho, L.F.; Romano, C.C. Application of PCR in serum samples for diagnosis of paracoccidioidomycosis in the southern Bahia-Brazil. *PLoS Negl Trop Dis* **2012**, *6*, e1909, doi:10.1371/journal.pntd.0001909.
10. Bialek, R.; Ibricevic, A.; Aepinus, C.; Najvar, L.K.; Fothergill, A.W.; Knobloch, J.; Graybill, J.R. Detection of *Paracoccidioides brasiliensis* in tissue samples by a nested PCR assay. *J Clin Microbiol* **2000**, *38*, 2940-2942.
11. Ricci, G.; Zelck, U.; Mota, F.; Lass-Florl, C.; Franco, M.F.; Bialek, R. Genotyping of *Paracoccidioides brasiliensis* directly from paraffin embedded tissue. *Med Mycol* **2008**, *46*, 31-34, doi:10.1080/13693780701488373.
12. Correia, J.; de Moraes Borba, C.; Reis, B.; Martins, A.; Unkles, S.; Kinghorn, J.R.; Lucena-Silva, N. The ceja-1 sequence as a potential new molecular marker for *Paracoccidioides brasiliensis* infection. *Mycoses* **2010**, *53*, 130-137, doi:10.1111/j.1439-0507.2008.01682.x.
13. Koishi, A.C.; Vituri, D.F.; Dionízio Filho, P.S.R.; Sasaki, A.A.; Felipe, M.S.S.; Venancio, E.J. A semi-nested PCR assay for molecular detection of *Paracoccidioides brasiliensis* in tissue samples. *Rev Soc Bras Med Trop* **2010**, *43*, 728-730.

14. Pitz Ade, F.; Koishi, A.C.; Tavares, E.R.; Andrade, F.G.; Loth, E.A.; Gandra, R.F.; Venancio, E.J. An optimized one-tube, semi-nested PCR assay for *Paracoccidioides brasiliensis* detection. *Rev Soc Bras Med Trop* **2013**, *46*, 783-785, doi:10.1590/0037-8682-1625-2013.
15. Gaviria, M.; Rivera, V.; Muñoz-Cadavid, C.; Cano, L.E.; Naranjo, T.W. Validation and clinical application of a nested PCR for paracoccidioidomycosis diagnosis in clinical samples from Colombian patients. *Braz J Infect Dis* **2015**, *19*, 376-383.
16. Arantes, T.D.; Theodoro, R.C.; Teixeira Mde, M.; Bosco Sde, M.; Bagagli, E. Environmental Mapping of *Paracoccidioides* spp. in Brazil Reveals New Clues into Genetic Diversity, Biogeography and Wild Host Association. *PLoS Negl Trop Dis* **2016**, *10*, e0004606, doi:10.1371/journal.pntd.0004606.
17. Nino-Vega, G.A.; Calcagno, A.M.; San-Blas, G.; San-Blas, F.; Gooday, G.W.; Gow, N.A. RFLP analysis reveals marked geographical isolation between strains of *Paracoccidioides brasiliensis*. *Med Mycol* **2000**, *38*, 437-441.
18. Roberto, T.N.; Rodrigues, A.M.; Hahn, R.C.; de Camargo, Z.P. Identifying *Paracoccidioides* phylogenetic species by PCR-RFLP of the alpha-tubulin gene. *Med Mycol* **2016**, *54*, 240-247, doi:10.1093/mmy/myv083.
19. Soares, C.M.; Madlun, E.E.; da Silva, S.P.; Pereira, M.; Felipe, M.S. Characterization of *Paracoccidioides brasiliensis* isolates by random amplified polymorphic DNA analysis. *J Clin Microbiol* **1995**, *33*, 505-507.
20. Calcagno, A.M.; Niño-Vega, G.; San-Blas, F.; San-Blas, G. Geographic discrimination of *Paracoccidioides brasiliensis* strains by randomly amplified polymorphic DNA analysis. *J Clin Microbiol* **1998**, *36*, 1733-1736, doi:10.1128/jcm.36.6.1733-1736.1998.
21. Molinari-Madlum, E.E.; Felipe, M.S.; Soares, C.M. Virulence of *Paracoccidioides brasiliensis* isolates can be correlated to groups defined by random amplified polymorphic DNA analysis. *Med Mycol* **1999**, *37*, 269-276.
22. Sano, A.; Tanaka, R.; Yokoyama, K.; Franco, M.; Bagagli, E.; Montenegro, M.R.; Mikami, Y.; Miyaji, M.; Nishimura, K. Comparison between human and armadillo *Paracoccidioides brasiliensis* isolates by random amplified polymorphic DNA analysis. *Mycopathologia* **1998**, *143*, 165-169, doi:10.1023/A:1006949113529.
23. Totti, D.O.; Romanha, A.J.; Grisard, E.C.; Simpson, A.J.; Koury, M.C. Random amplified polymorphic DNA (RAPD) analysis of *Paracoccidioides brasiliensis* isolates. *Rev Latinoam Microbiol* **1999**, *41*, 139-143.
24. Motta, T.R.; Moreira-Filho, C.A.; Mendes, R.P.; Souza, L.R.; Sugizak, M.F.; Baueb, S.; Calich, V.L.; Vaz, C.A. Evaluation of DNA polymorphisms amplified by arbitrary primers (RAPD) as genetically associated elements to differentiate virulent and non-virulent *Paracoccidioides brasiliensis* isolates. *FEMS Immunol Med Microbiol* **2002**, *33*, 151-157, doi:10.1111/j.1574-695X.2002.tb00585.x.
25. Hahn, R.C.; Macedo, A.M.; Santos, N.L.; Resende, J.C.; Hamdan, J.S. Characterization of *Paracoccidioides brasiliensis* atypical isolates by random amplified polymorphic DNA analysis. *Rev Iberoam Micol* **2002**, *19*, 49-51.
26. Hahn, R.C.; Macedo, A.M.; Fontes, C.J.; Batista, R.D.; Santos, N.L.; Hamdan, J.S. Randomly amplified polymorphic DNA as a valuable tool for epidemiological studies of *Paracoccidioides brasiliensis*. *J Clin Microbiol* **2003**, *41*, 2849-2854.
27. Batista Jr, J.; de Camargo, Z.P.; Fernandes, G.F.; Vicentini, A.P.; Fontes, C.J.F.; Hahn, R.C. Is the geographical origin of a *Paracoccidioides brasiliensis isolate* important for antigen production for regional diagnosis of paracoccidioidomycosis? *Mycoses* **2010**, *53*, 176-180, doi:10.1111/j.1439-0507.2008.01687.x.

28. Endo, S.; Komori, T.; Ricci, G.; Sano, A.; Yokoyama, K.; Ohori, A.; Kamei, K.; Franco, M.; Miyaji, M.; Nishimura, K. Detection of gp43 of *Paracoccidioides brasiliensis* by the loop-mediated isothermal amplification (LAMP) method. *FEMS Microbiol Lett* **2004**, *234*, 93-97, doi:10.1016/j.femsle.2004.03.015.
29. Tatibana, B.T.; Sano, A.; Uno, J.; Kamei, K.; Igarashi, T.; Mikami, Y.; Miyaji, M.; Nishimura, K.; Itano, E.N. Detection of *Paracoccidioides brasiliensis* gp43 gene in sputa by loop-mediated isothermal amplification method. *J Clin Lab Anal* **2009**, *23*, 139-143, doi:10.1002/jcla.20304.
30. Carvajal, D.M.L. Desenvolvimento de método diagnóstico através do desenho de sondas para a identificação de *Paracoccidioides lutzii*, utilizando a técnica de loop-mediated isothermal amplification (LAMP). Universidade estadual de Campinas Campinas., 2018.
31. Semighini, C.P.; de Camargo, Z.P.; Puccia, R.; Goldman, M.H.; Goldman, G.H. Molecular identification of *Paracoccidioides brasiliensis* by 5' nuclease assay. *Diagn Microbiol Infect Dis* **2002**, *44*, 383-386, doi:10.1016/s0732-8893(02)00472-8.
32. Buitrago, M.J.; Merino, P.; Puente, S.; Gomez-Lopez, A.; Arribi, A.; Zancope-Oliveira, R.M.; Gutierrez, M.C.; Rodriguez-Tudela, J.L.; Cuenca-Estrella, M. Utility of real-time PCR for the detection of *Paracoccidioides brasiliensis* DNA in the diagnosis of imported paracoccidioidomycosis. *Med Mycol* **2009**, *47*, 879-882, doi:10.3109/13693780802713208.
33. Theodoro, R.C.; Teixeira, M.d.M.; Felipe, M.S.S.; Paduan, K.d.S.; Ribolla, P.M.; San-Blas, G.; Bagagli, E. Genus *Paracoccidioides*: Species recognition and biogeographic aspects. *PLoS ONE* **2012**, *7*, e37694, doi:10.1371/journal.pone.0037694.
34. Rocha-Silva, F.; Gomes, L.I.; Gracielle-Melo, C.; Goes, A.M.; Caligiorne, R.B. Real Time Polymerase Chain Reaction (rt-PCR): A New Patent to Diagnostic Purposes for Paracoccidioidomycosis. *Recent Pat Endocr Metab Immune Drug Discov* **2017**, *10*, 143-149, doi:10.2174/1872214810666160905150958.
35. Costa, M.V.; Landgraf, T.N.; Corrêa, P.C.; Souza, I.E.L.; Fernandes, F.F.; Panunto-Castelo, A. Quantitation of pulmonary fungal burden in *Paracoccidioides brasiliensis*-infected mice by real-time PCR. *Rev Inst Med Trop Sao Paulo* **2019**, *61*.
36. De Brito, T.; Sandhu, G.S.; Kline, B.C.; Aleff, R.A.; Sandoval, M.P.; Santos, R.T.; Brandão, A.A.H.; Lacaz, C.S. In situ hybridization in paracoccidioidomycosis. *Med Mycol* **1999**, *37*, 207-211, doi:10.1080/j.1365-280X.1999.00222.x.
37. Arantes, T.D.; Theodoro, R.C.; Teixeira, M.d.M.; Bagagli, E. Use of fluorescent oligonucleotide probes for differentiation between *Paracoccidioides brasiliensis* and *Paracoccidioides lutzii* in yeast and mycelial phase. *Mem Inst Oswaldo Cruz* **2017**, *112*, 140-145.
38. Nobrega de Almeida, J., Jr.; Del Negro, G.M.; Grenfell, R.C.; Vidal, M.S.; Thomaz, D.Y.; de Figueiredo, D.S.; Bagagli, E.; Juliano, L.; Benard, G. Matrix-assisted laser desorption ionization-time of flight mass spectrometry for differentiation of the dimorphic fungal species *Paracoccidioides brasiliensis* and *Paracoccidioides lutzii*. *J Clin Microbiol* **2015**, *53*, 1383-1386, doi:10.1128/jcm.02847-14.
39. Comparato Filho, O.O.; Morais, F.V.; Bhattacharjee, T.; Castilho, M.L.; Raniero, L. Rapid identification of *Paracoccidioides lutzii* and *P. brasiliensis* using Fourier Transform Infrared spectroscopy. *J Mol Struct* **2019**, *1177*, 152-159, doi:https://doi.org/10.1016/j.molstruc.2018.09.016.
40. Alves, F.L.; Ribeiro, M.A.; Hahn, R.C.; de Melo Teixeira, M.; de Camargo, Z.P.; Cisalpino, P.S.; Marini, M.M. Transposable elements and two other molecular markers as typing tools for the genus *Paracoccidioides*. *Med Mycol* **2015**, *53*, 165-170, doi:10.1093/mmy/myu074.
41. Nascimento, E.; Martinez, R.; Lopes, A.R.; de Souza Bernardes, L.A.; Barco, C.P.; Goldman, M.H.S.; Taylor, J.W.; McEwen, J.G.; Nobrega, M.P.; Nobrega, F.G., et al. Detection and selection of

microsatellites in the genome of *Paracoccidioides brasiliensis* as molecular markers for clinical and epidemiological studies. *J Clin Microbiol* **2004**, *42*, 5007-5014, doi:10.1128/JCM.42.11.5007-5014.2004.

42. Matute, D.R.; Sepulveda, V.E.; Quesada, L.M.; Goldman, G.H.; Taylor, J.W.; Restrepo, A.; McEwen, J.G. Microsatellite analysis of three phylogenetic species of *Paracoccidioides brasiliensis*. *J Clin Microbiol* **2006**, *44*, 2153-2157, doi:10.1128/jcm.02540-05.
43. Turissini, D.A.; Gomez, O.M.; Teixeira, M.M.; McEwen, J.G.; Matute, D.R. Species boundaries in the human pathogen *Paracoccidioides*. *Fungal Genet Biol* **2017**, *106*, 9-25, doi:https://doi.org/10.1016/j.fgb.2017.05.007.
44. Matute, D.R.; McEwen, J.G.; Puccia, R.; Montes, B.A.; San-Blas, G.; Bagagli, E.; Rauscher, J.T.; Restrepo, A.; Morais, F.; Niño-Vega, G., et al. Cryptic speciation and recombination in the fungus *Paracoccidioides brasiliensis* as revealed by gene genealogies. *Mol Biol Evol* **2006**, *23*, 65-73, doi:10.1093/molbev/msj008.
45. Carrero, L.L.; Nino-Vega, G.; Teixeira, M.M.; Carvalho, M.J.; Soares, C.M.; Pereira, M.; Jesuino, R.S.; McEwen, J.G.; Mendoza, L.; Taylor, J.W., et al. New *Paracoccidioides brasiliensis* isolate reveals unexpected genomic variability in this human pathogen. *Fungal Genet Biol* **2008**, *45*, 605-612, doi:10.1016/j.fgb.2008.02.002.
46. White, T.J.; Bruns, T.; Lee, S.; Taylor, J. Amplification and direct sequencing of fungal ribosomal RNA genes for phylogenetics. In *PCR Protocols: A Guide to Methods and Applications*, Innis, M., Gelfand, D., Shinsky, J., White, T., Eds. Academic Press: New York, 1990; pp. 315-322.
47. Irinyi, L.; Lackner, M.; de Hoog, G.S.; Meyer, W. DNA barcoding of fungi causing infections in humans and animals. *Fungal Biol* **2015**, 10.1016/j.funbio.2015.04.007, doi: 10.1016/j.funbio.2015.1004.1007, doi:10.1016/j.funbio.2015.04.007.
48. Hebeler-Barbosa, F.; Montenegro, M.R.; Bagagli, E. Virulence profiles of ten *Paracoccidioides brasiliensis* isolates obtained from armadillos (*Dasypus novemcinctus*). *Med Mycol* **2003**, *41*, 89-96.
